# Characterization of midostaurin as a dual inhibitor of FLT3 and SYK and potentiation of FLT3 inhibition against FLT3-ITD-driven leukemia harboring activated SYK kinase

**DOI:** 10.18632/oncotarget.19036

**Published:** 2017-07-06

**Authors:** Ellen L. Weisberg, Alexandre Puissant, Richard Stone, Martin Sattler, Sara J. Buhrlage, Jing Yang, Paul W. Manley, Chengcheng Meng, Michael Buonopane, John F. Daley, Suzan Lazo, Renee Wright, David M. Weinstock, Amanda L. Christie, Kimberly Stegmaier, James D. Griffin

**Affiliations:** ^1^ Department of Medical Oncology, Dana-Farber Cancer Institute, Boston, Massachusetts, USA; ^2^ Department of Pediatric Oncology, Dana-Farber Cancer Institute, Boston, Massachusetts, USA; ^3^ Department of Cancer Biology, Dana-Farber Cancer Institute, Boston, Massachusetts, USA; ^4^ Department of Biological Chemistry and Molecular Pharmacology, Boston, Massachusetts, USA; ^5^ Novartis Institutes of Biomedical Research, Basel, Switzerland; ^6^ Department of Medicine, Harvard Medical School, Boston, Massachusetts, USA

**Keywords:** acute myelogenous leukemia, midostaurin, R406, R788, FLT3-ITD

## Abstract

Oncogenic FLT3 kinase is a clinically validated target in acute myeloid leukemia (AML), and both multi-targeted and selective FLT3 inhibitors have been developed. Spleen tyrosine kinase (SYK) has been shown to be activated and increased in FLT3-ITD-positive AML patients, and has further been shown to be critical for transformation and maintenance of the leukemic clone in these patients. Further, over-expression of constitutively activated SYK causes resistance to highly selective FLT3 tyrosine kinase inhibitors (TKI). Up to now, the activity of the multi-targeted FLT3 inhibitor, midostaurin, against cells expressing activated SYK has not been explored in the context of leukemia, although SYK has been identified as a target of midostaurin in systemic mastocytosis. We compared the ability of midostaurin to inhibit activated SYK in mutant FLT3-positive AML cells with that of inhibitors displaying dual SYK/FLT3 inhibition, targeted SYK inhibition, and targeted FLT3 inhibition. Our findings suggest that dual FLT3/SYK inhibitors and FLT3-targeted drugs potently kill oncogenic FLT3-transformed cells, while SYK-targeted small molecule inhibition displays minimal activity. However, midostaurin and other dual FLT3/SYK inhibitors display superior anti-proliferative activity when compared to targeted FLT3 inhibitors, such as crenolanib and quizartinib, against cells co-expressing FLT3-ITD and constitutively activated SYK-TEL. Interestingly, additional SYK suppression potentiated the effects of dual FLT3/SYK inhibitors and targeted FLT3 inhibitors against FLT3-ITD-driven leukemia, both in the absence and presence of activated SYK. Taken together, our findings have important implications for the design of drug combination studies in mutant FLT3-positive patients and for the design of future generations of FLT3 inhibitors.

## INTRODUCTION

Around 30% of patients with acute myeloid leukemia (AML) harbor activating mutations in *FLT3* [1], a gene normally involved in regulating hematopoiesis. The most common type of *FLT3* mutation results in internal tandem duplications (ITD) within the juxtamembrane domain, occurring in 20-25% of AML and strongly associated with decreased survival [2,3]. An additional 7% of patients have point mutations within the “activation loop” of *FLT3* [4].

Numerous FLT3 kinase inhibitors, both multi-targeted and selective, have been developed [5]. The N-indolocarbazole, midostaurin (PKC412; N-benzoyl-staurosporine; Novartis Pharma AG) was shown to target oncogenic FLT3 in preclinical studies [6] was reported to significantly prolong survival of FLT3-mutated AML patients when combined with conventional induction and consolidation therapies in a randomized Phase III clinical trial [7]. Midostaurin was recently FDA approved for treatment of adult, newly diagnosed AML patients positive for oncogenic FLT3, in combination with standard chemotherapy. Other FLT3 inhibitors in clinical development include quizartinib (AC220), which exhibits high potency and selectivity against FLT3-ITD [8], and crenolanib besylate (CP-868596; AROG Pharmaceuticals, LLC) [9].

Spleen tyrosine kinase (SYK) is an obligatory signaling partner for FLT3 that is required for transformation to AML and necessary for myeloproliferative disease (MPD) development [10]. SYK (wild-type (wt)) is expressed in most hematopoietic cells [11, 12] and belongs to the SYK/ZAP-70 family of non-receptor tyrosine kinases [13,14]. Oncogenic SYK has been identified as an important driver of different hematologic malignancies, including B-cell lymphoma, chronic lymphocytic leukemia (CLL) and mantle cell lymphoma [15–17], and was identified as a target in AML with SYK inhibition exhibiting anti-leukemia activity in mouse models of AML [18]. ITK-SYK, which results from the fusion between SYK and ITK (IL-2-inducible T-cell kinase), occurs as a recurrent translocation in 17% of patients with unspecified peripheral T-cell lymphomas [19,20]. TEL-SYK was originally detected in a patient with an atypical myelodysplastic syndrome with leukemic transformation [21,22]. TEL-SYK over-expression in murine pre B cells causes a B-acute lymphocytic leukemia (ALL)-like disease in mice [23] and cytoplasmic TEL-SYK fusion induces an acute panmyelosis with myelofibrosis-type acute myeloid leukemia (AML) in a bone marrow transplantation model [24].

Importantly, highly activated SYK has been found to be enriched in AML patients with a higher frequency in patients harboring the *ITD* mutant than wild-type (wt) *FLT3* [10]. SYK has also been shown to be associated with resistance to FLT3-ITD-targeted therapy, and inhibition of FLT3-ITD with quizartinib combined with inhibition of SYK was shown to be more effective than FLT3 inhibition alone in FLT3-ITD-positive models of AML [10].

Given the reported significance of SYK in transformation and maintenance of AML, as well as FLT3 kinase inhibitor resistance, and implications of SYK as a potentially important target for AML treatment, we were interested in investigating the ability of midostaurin to inhibit SYK in FLT3 mutant-positive AML. SYK has previously been shown to be a target of midostaurin and its metabolites, CGP52421 and CGP62221, in advanced systemic mastocytosis, a hematopoietic neoplasm characterized by expansion and abnormal accumulation of mast cells [25]. Up to now, however, the activity of midostaurin, alone and combined with SYK inhibition, against cells expressing activated SYK has not been explored in the context of leukemia.

Here, we compare the SYK-targeting activity of midostaurin with different classes of kinase inhibitors, including those with SYK inhibitory activity and those without. We show that midostaurin, like the dual FLT3/SYK inhibitors, R406 (tamatinib), and R788 (fostamatinib) [26], inhibits SYK in cell-based models of FLT3-ITD- and activated SYK-driven leukemia to a greater extent than highly targeted inhibitors of FLT3. We also show that SYK inhibition alone is not sufficient to kill FLT3-ITD-positive cells and that the SYK inhibitory activity of midostaurin is insufficient to potently kill cells expressing activated SYK. However, additional targeted SYK inhibition or dual FLT3/SYK inhibition potentiates the effects of midostaurin and other inhibitors of FLT3 against both kinase inhibitor-sensitive- and -resistant FLT3-ITD- and activated SYK-positive leukemia.

## RESULTS

### SYK is a target of midostaurin

As a first assessment of the ability of midostaurin to inhibit SYK we tested its inhibitory activity of SYK in a purified enzyme assay and found the compound inhibits SYK with an IC_50_ of 20.8 nM. In similar enzyme assays, the SYK inhibitor, R788, and its active metabolite, R406, have been determined to inhibit SYK with an IC_50_ of 41 nM [26]. Both compounds are reported to be 5-fold less potent against FLT3 than Syk. Both R788 and R406 have been evaluated in clinical trials. In comparison, a highly targeted SYK inhibitor, PRT062607 (P505, BIIB057) has been reported in cell-free assays to inhibit SYK with an IC_50_ of 1 nM and to inhibit FLT3 with an IC_50_ of 139 nM [27].

### Effects of midostaurin, R406, and R788 on Ba/F3 cells expressing activated SYK

**Figure 1:**
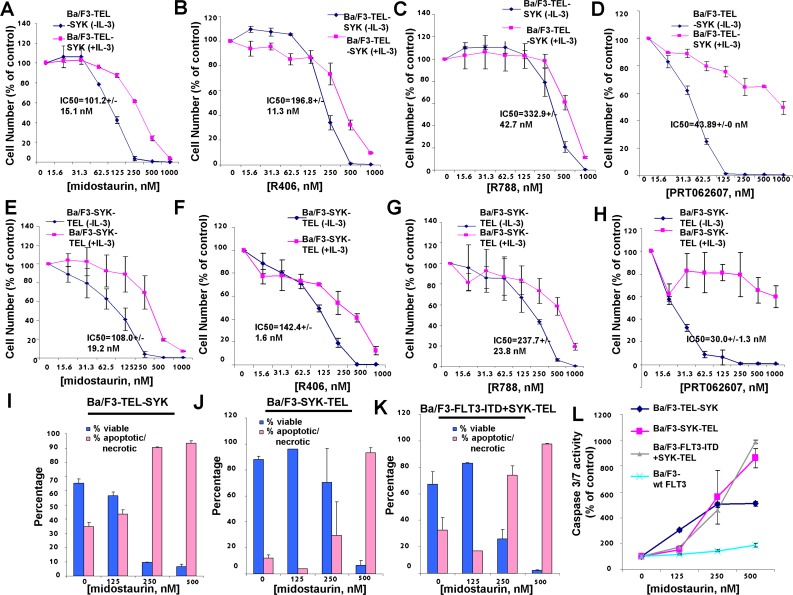
Effects of midostaurin, R406, R788, and PRT062607 on inhibition of proliferation of Ba/F3 cells expressing constitutively activated TEL-SYK or constitutively activated SYK-TEL **A**.-**D**. Three-day treatment of Ba/F3-TEL-SYK cells with midostaurin (A), R406 (B), R788 (C), or PRT062607 (D) in the presence and absence of 20% WEHI (used as a source of IL-3). **E**.-**H**. Three-day treatment of Ba/F3-SYK-TEL cells with midostaurin (E), R406 (F), R788 (G), or PRT062607 (H). Error bars represent the mean+/−S.D. for all proliferation studies. **I**.-**K**. Effects of midostaurin on apoptosis (annexin/pi measurement) in cells expressing active SYK. Error bars represent the mean+/−S.D. **L**. Effects of midostaurin on apoptosis (caspase 3/7 measurement) in cells expressing active SYK. Error bars represent the mean+/−S.D.

In order to investigate the ability of midostaurin to inhibit SYK activity in cells, we utilized Ba/F3 cell lines that stably express constructs encoding fusion proteins comprised of SYK kinase coupled with a TEL moiety that constitutively activates SYK [10] (Supplementary Figure 1). Specifically, one Ba/F3 cell line was developed to over-express constitutively active “TEL-SYK,” the protein product of a fusion between a truncated form of *SYK* that is devoid of its SH2 domains, SH2 Nter + SH2 Cter, and a *TEL* sequence that takes the place of these two domains [22, 10]. A second Ba/F3 cell line tested was engineered to over-express a constitutively and highly active variant of SYK, “SYK-TEL,” which, unlike TEL-SYK, contains an intact SH2 domain (Supplementary Figure 1). Consistent with the order of potencies generated in cell-free assays, midostaurin inhibited the growth of Ba/F3-TEL-SYK cells with an IC_50_ of 101.2 nM, a 2- to 3-fold higher potency than that of R406 (IC_50_ = 196.8 nM) and R788 (IC_50_ = 332.9 nM), however a lower potency than the IC_50_ of 43.8 nM for PRT062607 against these cells (Figure 1A-1D and Table 1A). The effects of midostaurin, R406, and R788 against Ba/F3-TEL-SYK cells were modestly IL-3 rescued; the effects of PRT062607 were highly IL-3 rescue-able.

**Table 1A:** IC50s (nM) (mean+/−S.D.), generated using GraphPad Prism 7 Software, for R406, midostaurin, R788, and PRT062607 tested against Ba/F3-SYK-TEL, Ba/F3-TEL-SYK, Ba/F3-FLT3-ITD, Ba/F3-FLT3-ITD+SYK-TEL, and Ba/F3-FLT3-ITD+TEL-SYK. Cell growth assays were carried out for approximately 3 days

	R406 (IC50, nM)	midostaurin (IC50, nM)	R788 (IC50, nM)	PRT062607 (IC50, nM)
**Ba/F3-SYK-TEL**	142.4+/− 1.6	108.0+/−19.2	237.7+/−23.8	30.0+/−1.3
**Ba/F3-TEL-SYK**	196.8+/−11.3	101.2+/−15.1	332.9+/−42.7	43.8+/−0.1
**Ba/F3-FLT3-ITD**	36.7+/−20.5	6.3+/−3	61.5+/−18.9	1078.0+/−110.4
**Ba/F3-FLT3-ITD+SYK-TEL**	223.7+/−42.2	198.2+/−21.6	347.8+/−71.4	48.4+/−0.3
**Ba/F3-FLT3-ITD+TEL-SYK**	27.0+/−3.4	3.0+/−0.2	47.1+/−6.3	614.4

**Table 1B:** IC50s (nM) (mean+/−S.D.), generated using GraphPad Prism 7 Software, for crenolanib and AC220 tested against Ba/F3-FLT3-ITD and Ba/F3-FLT3-ITD+SYK-TEL. Cell growth assays were carried out for approximately 3 days

	crenolanib (IC50, nM)	quizartinib (IC50, nM)
**Ba/F3-FLT3-ITD**	6.2+/−2	0.3+/−0.3
**Ba/F3-FLT3-ITD+SYK-TEL**	520.6+/−121.1	627.5+/−32.2

Similar results were observed with midostaurin treatment of Ba/F3-SYK-TEL cells, with a higher potency exhibited by midostaurin (IC_50_ = 108.0 nM) as compared to R406 (IC_50_ = 142.4 nM) and R788 (IC_50_ = 237.7 nM), yet a lower potency as compared to PRT062607 (IC_50_ = 30.0 nM) (Figure 1E-1H and Table 1A). Midostaurin was observed to induce apoptosis, as measured by annexin/PI staining, in cells expressing constitutively activated SYK (Figure 1I-1K). Consistent with this, midostaurin also more robustly increased the activities of caspase-3 and -7 in activated SYK-expressing cells as compared to growth factor-dependent Ba/F3 cells over-expressing wt FLT3 (Figure 1L) and led to an increase in the subG0/G1 fraction as determined by PI staining (Supplementary Figure 2).

### Effects of midostaurin, R406, and R788 on Ba/F3 cells co-expressing FLT3-ITD and activated SYK

**Figure 2:**
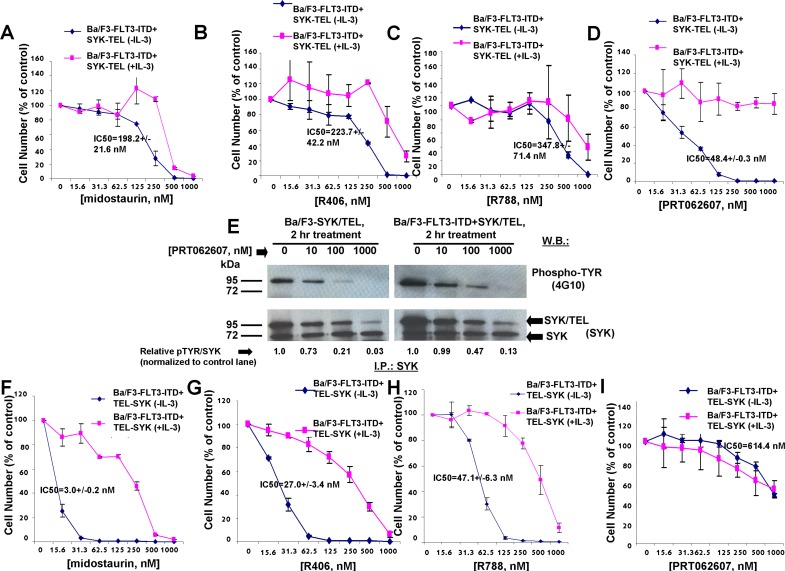
Effects of midostaurin, R406, R788, and PRT062607 on inhibition of proliferation of Ba/F3 cells expressing TEL-SYK or SYK-TEL co-expressed with FLT3-ITD **A**.-**D**. Approximately 3-day treatment of Ba/F3-FLT3-ITD+SYK-TEL cells with midostaurin (A), R406 (B), R788 (C), or PRT062607 (D) in the presence and absence of 20% WEHI (used as a source of IL-3). Error bars represent the mean+/−S.D. for all proliferation studies. **E**. Effect of PRT062607 on phosphorylation of SYK-TEL in Ba/F3-SYK-TEL and Ba/F3-FLT3-ITD+SYK-TEL cells. For this experiment, SYK immunoprecipitation was carried out prior to immunoblotting with a PTYR (4G10) antibody. ImageJ 32 software was used for densitometry. Briefly, to get band intensities, the area of all the bands was first measured, and then normalized to the control lane, and then normalized to total protein. **F**.-**I**. Approximately 3-day treatment of Ba/F3-FLT3-ITD+TEL-SYK cells with midostaurin (F), R406 (G), R788 (H), or PRT062607(I). Error bars represent the mean+/−S.D. for all proliferation studies.

Oncogenic FLT3 has been previously demonstrated to be phosphorylated by constitutively activated SYK through its SH2 domain, and co-expression of SYK-TEL and FLT3-ITD leads to an association between the two proteins and renders cells resistant to FLT3 kinase inhibition [10]. We were interested in comparing the activities of dual FLT3/SYK inhibitors, midostaurin, R406, and R788, and the selective SYK inhibitor, PRT062607, against Ba/F3-FLT3-ITD+SYK-TEL cells. Compared to drug effects on Ba/F3-SYK-TEL cells, a 1.5-1.8-fold loss of potency against Ba/F3-FLT3-ITD+SYK-TEL cells was observed for midostaurin (IC50=198.2 nM), R406 (IC50=223.7 nM), and R788 (IC50=347.8 nM), and PRT062607 (IC50=48.4 nM) (Figure 1E-1H, Figure 2A-2D, and Table 1A). Inhibition of growth of cells co-expressing FLT3-ITD and SYK-TEL by midostaurin correlated with induction of apoptosis (Figure 1K). Potency differences for the inhibitors against Ba/F3-SYK-TEL versus Ba/F3-FLT3-ITD+SYK-TEL cells were reflected in the ability of midostaurin and R406 to suppress the activity of signaling molecules downstream of SYK and FLT3 to a slightly higher extent in Ba/F3-SYK-TEL cells than Ba/F3-FLT3-ITD+SYK-TEL cells, including STAT5, AKT, and S6 (Supplementary Figure 3A-3B). Similarly, inhibition of SYK phosphorylation by PRT062607 in Ba/F3-SYK-TEL cells was more significant than in Ba/F3-FLT3-ITD+SYK-TEL cells (Figure 2E).

**Figure 3:**
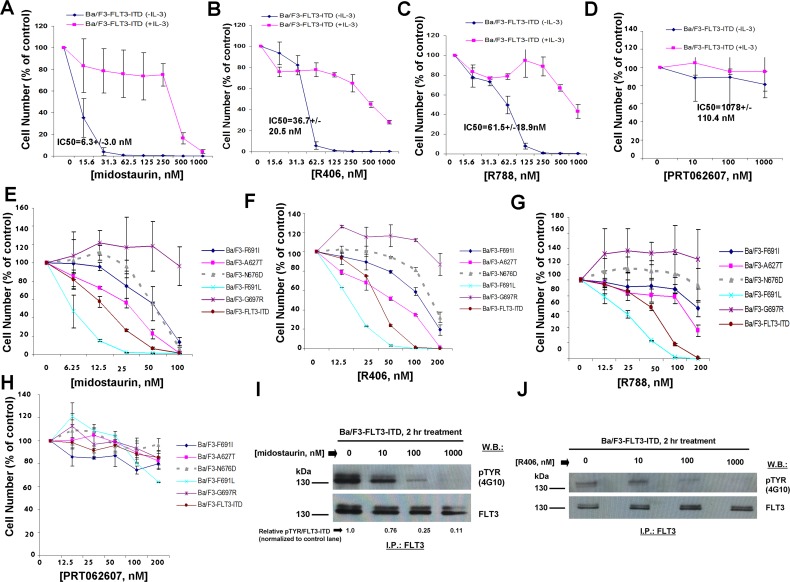
Effects of midostaurin, R406, R788, and PRT062607 on Ba/F3 cells expressing FLT3-ITD **A**.-**D**. Three-day treatment of Ba/F3-FLT3-ITD cells with midostaurin (A), R406 (B), R788 (C), or PRT062607 in the presence and absence of 20% WEHI (used as a source of IL-3). **E**.-**H**. Three-day treatment of kinase inhibitor-resistant FLT3-ITD+TKD point mutants with midostaurin (E), R406 (F), R788 (G), or PRT062607 (H). Error bars represent the mean+/−S.D. for all proliferation studies. **I**.-**J**. Effect of midostaurin (I) and R406 (J) on phosphorylation of FLT3-ITD. For this experiment, FLT3 immunoprecipitation was carried out prior to immunoblotting with a PTYR (4G10) antibody. ImageJ 32 software was used for densitometry. Briefly, to get band intensities, the area of all the bands was first measured, and then normalized to the control lane, and then normalized to total protein.

The SH2-binding region expressed on SYK-TEL but missing from TEL-SYK allows phosphorylation of FLT3 by SYK through a functional interaction between SYK and FLT3 [10] (Supplementary Figure 1). TEL-SYK, in contrast, despite being constitutively active, is not able to activate FLT3 to the extent of SYK-TEL [10] (Supplementary Figure 1). Midostaurin, R406, and R788 displayed high potencies against Ba/F3-FLT3-ITD+TEL-SYK cells, with IC50 values of 3.0 nM, 27 nM, and 47.1 nM, respectively (Figure 2F-2H and Table 1A), as compared to Ba/F3-FLT3-ITD+SYK-TEL cells. PRT062607, in contrast, displayed comparatively low potency against Ba/F3-FLT3-ITD+TEL-SYK cells (IC50 614.4 nM) (Figure 2I). Thus, although this line was included for investigation, the data suggest that it may be predominantly driven by FLT3-ITD. In support of this, the potencies of midostaurin, R406, R788, and PRT062607 against Ba/F3-FLT3-ITD+TEL-SYK were very similar to potencies of these compounds against Ba/F3-FLT3-ITD cells, with IC50 values of 6.3 nM, 36.7 nM, 61.5 nM, and 1078 nM, respectively (Figure 3A-3D and Table 1A).

For comparison with Ba/F3 cells expressing FLT3-ITD, we tested the effects of dual SYK/FLT3 and targeted SYK inhibitors against cells expressing FLT3-ITD and kinase inhibitor-resistant tyrosine kinase domain (TKD) point mutations. As expected, midostaurin, R406, and R788 treatment killed Ba/F3 cells expressing FLT3-ITD+FLT3 kinase inhibitor-resistant TKD point mutants to varying extents although each generally with less potency than toward FLT3-ITD, whereas PRT062607 was completely inactive against these cells (Figure 3E-3H).

**Figure 4:**
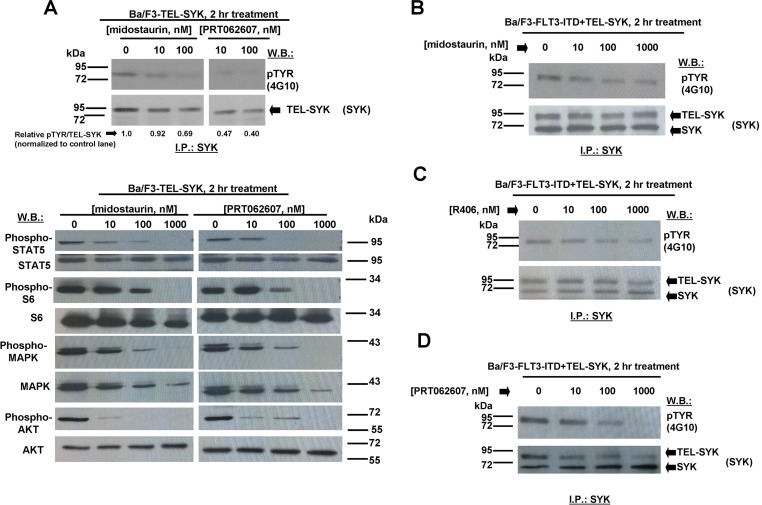
Effects of midostaurin, R406, and PRT062607 on phosphorylation of constitutively activated TEL-SYK and downstream signaling molecules **A**. Effect of midostaurin and PRT062607 on TEL-SYK phosphorylation and phosphorylation of downstream effectors following 2 hr of treatment. For the experiment analyzing effects of midostaurin and PRT062607 on phosphorylation of TEL-SYK, SYK immunoprecipitation was carried out prior to immunoblotting with a PTYR (4G10) antibody. Immunoblotting was performed for the experiment analyzing effects of midostaurin and PRT062607 on signaling molecules downstream of TEL-SYK. Relative densitometry readings for pMAPK/total MAPK (normalized to control lane) are as follows: 0 nM midostaurin=1.0, 10 nM midostaurin=0.93, 100 nM midostaurin=0.41, 1000 nM midostaurin=0.04, 0 nM PRT062607=1.0, 10 nM PRT062607=0.72, 100 nM PRT062607=0.37, 1000 nM PRT062607=0.07. **B**.-**D**. Effects of midostaurin, R406, and PRT062607 on TEL-SYK phosphorylation in Ba/F3 cells co-expressing FLT3-ITD and TEL-SYK. For these experiments, SYK immunoprecipitation was carried out prior to immunoblotting with a PTYR (4G10) antibody. ImageJ 32 software was used for densitometry. Briefly, to get band intensities, the area of all the bands was first measured, and then normalized to the control lane, and then normalized to total protein.

We next explored the ability of midostaurin, R406, and PRT062607 to inhibit the phosphorylation of FLT3 or SYK in Ba/F3 cells transformed by activated versions of each. Midostaurin and R406 were effective at inhibiting FLT3 phosphorylation in a concentration-dependent manner in Ba/F3-FLT3-ITD cells, which correlated with induction of cell death by these compounds (Figure 3I-3J). In Ba/F3-TEL-SYK cells, midostaurin, similar to PRT062607, inhibited TEL-SYK phosphorylation and activity of downstream effectors of TEL-SYK across a concentration range that led to inhibition of cell growth by the compounds (Figure 4A). In Ba/F3-FLT3-ITD+TEL-SYK cells, both midostaurin and R406 inhibited phosphorylation of TEL-SYK, although to a lesser extent than PRT062607 (Figure 4B-4D). Despite the observed inhibitory activity of PRT062607 against TEL-SYK, the fact that PRT062607 is a comparatively weak inhibitor of Ba/F3-FLT3-ITD+TEL-SYK proliferation again supports the notion that Ba/F3-FLT3-ITD+TEL-SYK cells are predominantly driven by FLT3-ITD.

**Figure 5:**
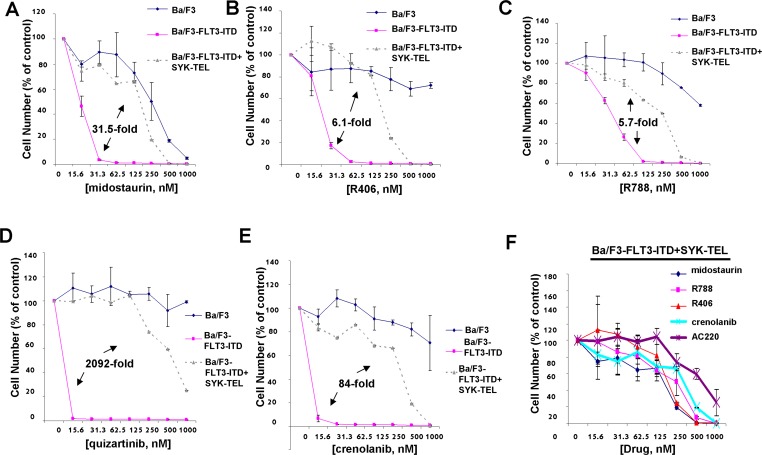
Comparison of effects of targeted FLT3 inhibitors with dual SYK/FLT3 inhibitors on cells expressing FLT3-ITD and activated SYK **A**.-**E**. Three-day treatment of Ba/F3, Ba/F3-FLT3-ITD, and Ba/F3-FLT3-ITD+SYK-TEL cells with midostaurin (A), R406 (B), R788 (C), quizartinib (D), or crenolanib (E). **F**. Comparison of effects of dual SYK/FLT3 inhibitors versus selective FLT3 inhibitors on growth of Ba/F3 cells co-expressing highly activated SYK-TEL and FLT3-ITD following 3 days of treatment. Error bars represent the mean+/−S.D. for all proliferation studies. Fold differences between IC50s for inhibitor treatment of Ba/F3-FLT3-ITD and Ba/F3-FLT3-ITD+SYK-TEL are shown for all of the graphs. Parental Ba/F3 cells are shown as a control for nonspecific toxicity.

Midostaurin, R406, R788, quizartinib, and crenolanib were tested in parallel against parental Ba/F3 cells (as a control for nonspecific toxicity), Ba/F3-FLT3-ITD, and Ba/F3-FLT3-ITD+SYK-TEL to compare the anti-proliferative effects of inhibitors targeting both FLT3-ITD and SYK (midostaurin, R406, R788) and those targeting FLT3-ITD alone (quizartinib, crenolanib) (Figure 5). Compared to IC_50_s generated for midostaurin (6.3 nM), R406 (36.7nM), and R788 (61.5 nM) against Ba/F3-FLT3-ITD cells, treatment of Ba/F3-FLT3-ITD+SYK-TEL cells with midostaurin (198.2 nM), R406 (223.7 nM), or R788 (347.8 nM) led to an average 31.5-fold shift for midostaurin, 6.1-fold shift for R406, and 5.7-fold shift for R788 (Figure 5A-5C). In contrast, compared to the IC_50_s for quizartinib (0.3 nM) or crenolanib (6.2 nM) against Ba/F3-FLT3-ITD cells, treatment of Ba/F3-FLT3-ITD+SYK-TEL cells with quizartinib (627.5 nM) or crenolanib (520.6 nM) led to an average 2092-fold higher IC_50_ for quizartinib and an 84-fold higher for crenolanib (Figure 5D-5E). Graphing of drug effects against Ba/F3-FLT3-ITD+SYK-TEL cells together shows less responsiveness of these cells to targeted FLT3 inhibitors, such as crenolanib and quizartinib, both of which lack SYK as a target, compared to midostaurin, R788, and R406 (Figure 5F). These results suggest the importance of a FLT3 kinase inhibitor also possessing SYK inhibitory activity in order to more potently kill leukemia cells characterized as expressing both mutant FLT3 and activated SYK.

It is important to note that concentrations of R406, and R788 were relatively ineffective against parental Ba/F3 cells at up to 500-1000 nM following three days of drug treatment, however concentrations of midostaurin over 250 nM led to anti-proliferative effects against parental Ba/F3 cells suggesting that nonspecific toxicity could occur at those levels of drug (Figure 5). In contrast, PRT062607 was ineffective against parental Ba/F3 cells at up to 1000 nM (Supplementary Figure 4).

### Potentiation of the anti-leukemic effects of midostaurin by dual FLT3/SYK suppression and targeted SYK suppression

The ability of midostaurin to synergize with dual FLT3/SYK inhibitors, R406 and R788, or the highly targeted SYK inhibitor, PRT062607, was tested in a panel of Ba/F3 cell lines expressing constitutively active SYK in the presence or absence of FLT3-ITD, as well as Ba/F3-FLT3-ITD cells and human AML lines, such as MOLM13-luc+, MOLM14, and MV4,11, driven by FLT3-ITD. As described in detail below, general observations from these experiments include potentiation of the anti-proliferative effects of midostaurin by inhibitors targeting SYK, which supports the notion that additional SYK suppression is of benefit against FLT3-ITD-driven AML treated with a FLT3 kinase inhibitor.

**Figure 6:**
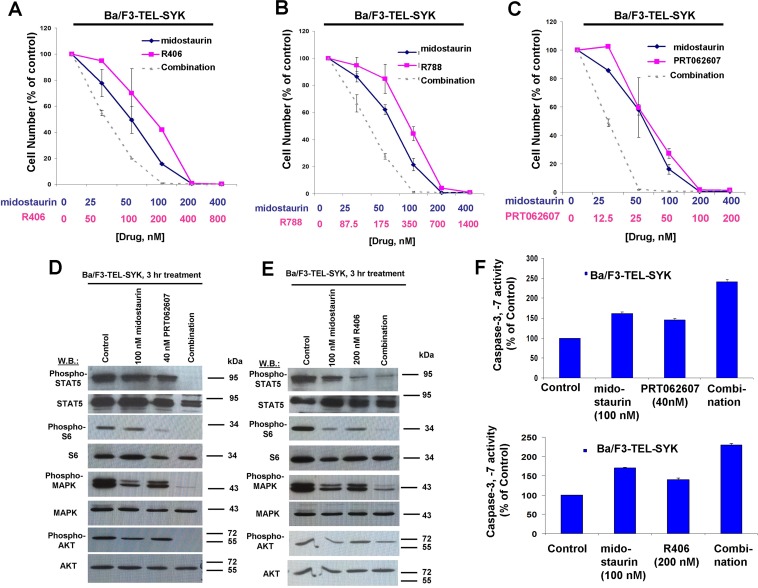
Potentiation of effects of midostaurin against Ba/F3-TEL-SYK cells by R406, R788, and PRT062607 **A**.-**C**. Three-day treatments of Ba/F3-TEL-SYK cells with midostaurin+/−R406, midostaurin+/−R788, or midostaurin+/−PRT062607. Error bars represent the mean+/−S.D. for all proliferation studies. **D**. Effects of midostaurin (100 nM), PRT062607 (40 nM), or a combination on signaling molecules downstream of TEL-SYK. **E**. Effects of midostaurin (100 nM), R406 (200 nM), or a combination on signaling molecules downstream of TEL-SYK. Immunoblotting was performed for the experiment analyzing effects of midostaurin+/−PRT062607 and midostaurin+/−R406 on signaling molecules downstream of TEL-SYK. **F**. Caspase activity in Ba/F3-TEL-SYK cells treated with midostaurin +/− PRT062607 or midostaurin +/− R406.

**Figure 7:**
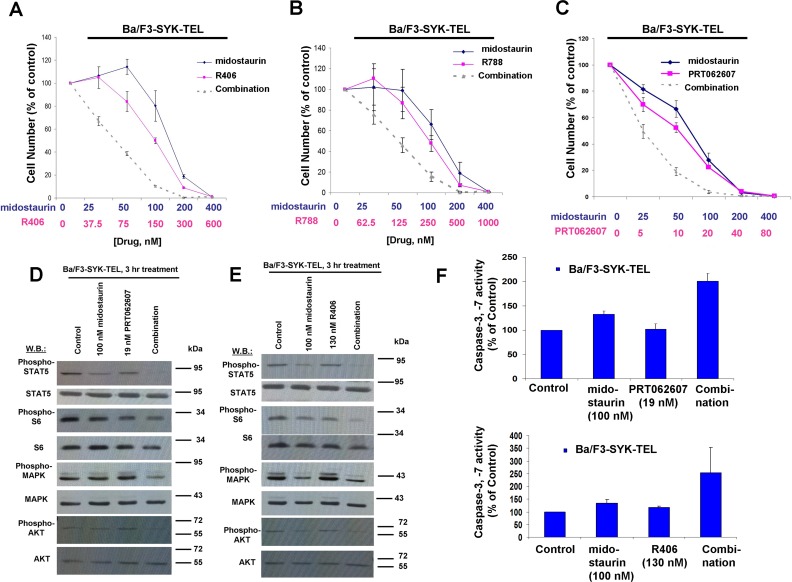
Potentiation of effects of midostaurin against Ba/F3-SYK-TEL cells by R406, R788, and PRT062607 **A**.-**C**. Three-day treatments of Ba/F3-SYK-TEL cells with midostaurin+/−R406, midostaurin+/−R788, or midostaurin+/−PRT062607. **D**. Effects of midostaurin (100 nM), PRT062607 (19 nM), or a combination on signaling molecules downstream of SYK-TEL. Error bars represent the mean+/−S.D. for all proliferation studies. **E**. Effects of midostaurin (100 nM), R406 (130 nM), or a combination on signaling molecules downstream of SYK-TEL. Immunoblotting was performed for the experiment analyzing effects of midostaurin+/−PRT062607 and midostaurin+/−R406 on signaling molecules downstream of SYK-TEL. **F**. Caspase activity in Ba/F3-SYK-TEL cells treated with midostaurin +/− PRT062607 or midostaurin +/− R406.

**Figure 8:**
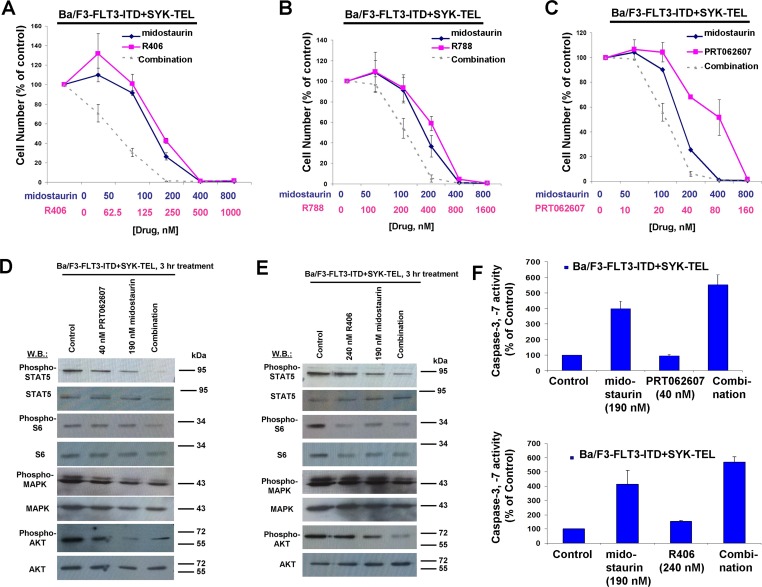
Potentiation of effects of midostaurin against Ba/F3-FLT3-ITD+SYK-TEL cells by R406, R788, and PRT062607 **A**.-**C**. Three-day treatments of Ba/F3-FLT3-ITD+SYK-TEL cells with midostaurin+/−R406, midostaurin+/−R788, or midostaurin+/−PRT062607. **D**. Effects of midostaurin (190 nM), PRT062607 (40nM), or a combination on signaling molecules downstream of Ba/F3-FLT3-ITD+SYK-TEL. Error bars represent the mean+/−S.D. for all proliferation studies. **E**. Effects of midostaurin (190 nM), R406 (240 nM), or a combination on signaling molecules downstream of Ba/F3-FLT3-ITD+SYK-TEL. Immunoblotting was performed for the experiment analyzing effects of midostaurin+/−PRT062607 and midostaurin+/−R406 on signaling molecules downstream of FLT3-ITD+SYK-TEL. **F**. Caspase activity in Ba/F3-FLT3-ITD+SYK-TEL cells treated with midostaurin +/− PRT062607 or midostaurin +/− R406.

Concentration ranges tested for the different compounds alone and in combination were based on their estimated cellular IC_50_ values. Effects of midostaurin against Ba/F3-TEL-SYK, Ba/F3-SYK-TEL, Ba/F3-FLT3-ITD+TEL-SYK, and Ba/F3-FLT3-ITD+SYK-TEL were potentiated to varying extents by R788, R406 and PRT062607, with additive to synergistic interactions observed (Figures 6A-6C, 7A-7C, 8A-8C, Supplementary Figure 5 and Table 2). Combination effects of midostaurin+/−R406 and midostaurin+/−PRT062607 that led to increased inhibition of proliferation of constitutively activated SYK-expressing cells correlated with increased suppression of phosphorylation of downstream effectors of SYK, including STAT5, S6, MAPK, and AKT (Figures 6D-6E, 7D-7E, 8D-8E). Combination effects of these drug combinations also correlated with increased caspase-3 and -7 activity, suggesting increased apoptosis in drug combination-treated cells (Figures 6-8F).

**Figure 9:**
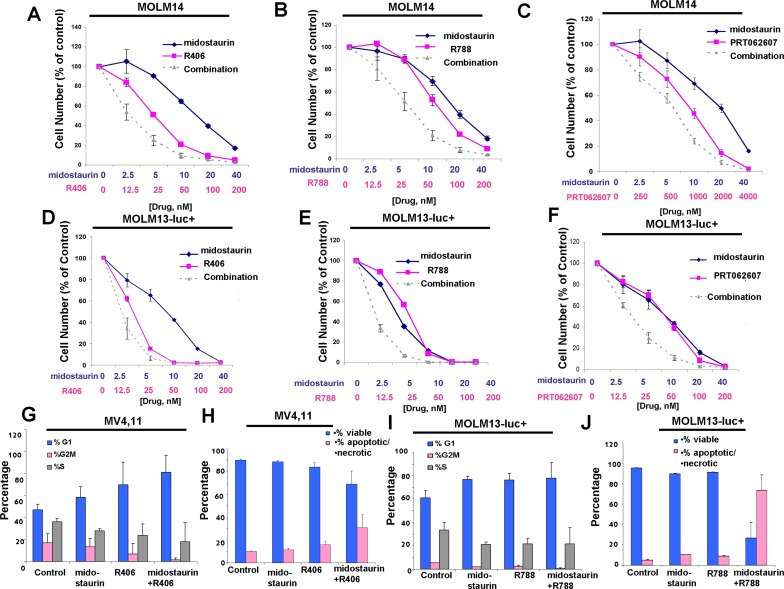
Potentiation of midostaurin by R406, R788 and PRT062607 against FLT3-ITD-driven cell lines **A**.-**C**. Three-day treatments of MOLM14 cells with midostaurin+/−R406, midostaurin+/−R788, or midostaurin+/−PRT062607. **D**.-**F**. Three-day treatments of MOLM13-luc+ cells with midostaurin+/−R406, midostaurin+/−R788, or midostaurin+/−PRT062607. Error bars represent the mean+/−S.D. for all proliferation studies. **G**.-**H**. Effects of midostaurin (10 nM) +/−R406 (50 nM) on MV4,11 cell cycle progression (G) and apoptosis (H). **I**.-**J**. Effects of midostaurin (10 nM) +/− R788 (50 nM) on MOLM13-luc+ cell cycle progression (I) and apoptosis (J). Error bars represent the mean+/−S.D.

The combination of midostaurin+/−R406, midostaurin+/−R788, and midostaurin+/−PRT062607 also led to increased inhibition of the growth of Ba/F3-FLT3-ITD cells (Supplementary Figure 6A-C). Importantly, combination effects observed were confirmed not to be unique to the Ba/F3 system. Positive combination effects were also observed between midostaurin +/− R406, R788, or PRT062607 against human FLT3-ITD-driven MOLM13, MOLM14, and MV411 cells (Figure 9A-9F, Supplementary Figure 6D-6F and Table 2). Increases in G1 arrest and/or percent of apoptotic cells and fraction of cells in subG0/G1 were observed for FLT3-ITD-driven cells treated with combinations of midostaurin and R406 or R788 (Figure 9G-9J and Supplementary Figure 7). It should be noted that, in contrast to drug combinations against other cell lines, generally no additive to synergistic effects were observed for midostaurin and PRT062607 against the MV4,11 cell line (Supplementary Figure 6F).

**Table 2:** Combination indices generated by Calcusyn software for midostaurin+/− R406, R788, and PRT062607 tested against Ba/F3-SYK-TEL, Ba/F3-TEL-SYK, Ba/F3-FLT3-ITD, Ba/F3-FLT3-ITD+SYK-TEL, Ba/F3-FLT3-ITD+TEL-SYK, MOLM14, MOLM13- luc+, MV4,11, and midostaurin-resistant MOLM13-luc+ cells

Cell Line, Drug Treatments	ED25	ED50	ED75	ED90	
**Ba/F3-SYK-TEL**					
midostaurin+R406	0.39943	0.51171	0.65589	0.84114	
midostaurin+R788	0.52255	0.65469	0.82026	1.02774	
midostaurin+PRT062607	0.80590	0.87952	0.96109	1.05159	
**Ba/F3-TEL-SYK**					
midostaurin+R406	0.76376	0.85078	0.94864	1.05881	
midostaurin+R788	0.73959	0.82537	0.92131	1.0286	
midostaurin+PRT062607	0.13909	0.21598	0.33684	0.52773	
**Ba/F3-FLT3-ITD**					
midostaurin+R406	0.74152	0.74592	0.75824	0.77845	
midostaurin+R788	0.47348	0.53233	0.60096	0.68133	
midostaurin+PRT062607	0.54961	0.54046	0.53808	0.54124	
**Ba/F3-FLT3-ITD+SYK-TEL**					
midostaurin+R406	0.31570	0.43963	0.61232	0.85301	
midostaurin+R788	0.85128	0.99349	1.15950	1.35328	
midostaurin+PRT062607	0.86375	0.97146	1.09260	1.22885	
**Ba/F3-FLT3-ITD+TEL-SYK**					
midostaurin+R406	0.73305	0.82401	0.92631	1.04135	
midostaurin+R788	0.77081	0.84045	0.91657	0.99981	
midostaurin+PRT062607	0.48694	0.60149	0.74306	0.91802	
**MOLM14**					
midostaurin+R406	0.42615	0.54750	0.71916	0.96943	
midostaurin+R788	0.55385	0.66601	0.82207	1.04052	
midostaurin+PRT062607	0.88142	0.92061	0.96929	1.02933	
**MOLM13-luc+**					
midostaurin+R406	0.28109	0.39832	0.56512	0.80281	
midostaurin+R788	0.46901	0.55688	0.66214	0.78838	
midostaurin+PRT062607	0.75206	0.84920	0.96037	1.08776	
**MV411**					
midostaurin+R406	0.85296	0.85000	0.84890	0.84963	
midostaurin+R788	0.74899	0.78910	0.87103	1.00545	
midostaurin+PRT062607	1.28030	1.18799	1.10918	1.04150	
**midostaurin-resistant MOLM13-luc+**					
midostaurin+R406	0.94762	1.00537	1.06943	1.14050	
midostaurin+R788	1.16299	1.09052	1.02404	0.96302	
midostaurin+PRT062607	0.46223	0.59674	0.77041	0.99465	
**Ba/F3-FLT3-ITD**					
crenolanib+PRT062607	1.06052	1.09187	1.12443		1.15826
**Ba/F3-FLT3-ITD+SYK-TEL**					
crenolanib+PRT062607	0.93074	0.93282	0.94592		0.97158

Also shown are combination indices generated by Calcusyn software for crenolanib+/−PRT062607 tested agianst Ba/F3-FLT3-ITD and Ba/F3-FLT3-ITD+SYK-TEL. Proliferation assays/combination studies were carried out for approximately 3 days.

**Figure 10:**
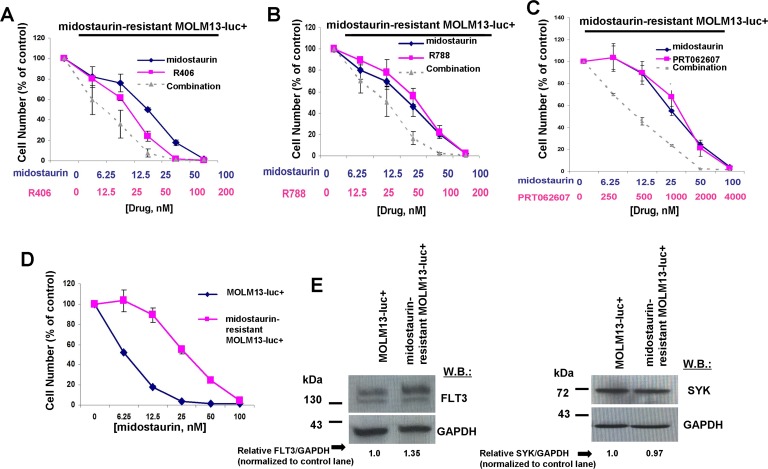
Potentiation of effects of midostaurin against FLT3 inhibitor-resistant cells by R406, R788, and PRT062607 **A**.-**C**. Three-day treatments of midostaurin-resistant MOLM13-luc+ cells with midostaurin+/−R406, midostaurin+/−R788, or midostaurin+/−PRT062607. **D**. Three-day treatment of MOLM13-luc+ and midostaurin-resistant MOLM13-luc+ cells with midostaurin. Error bars represent the mean+/−S.D. for all proliferation studies. **E**. Comparison of FLT3 and SYK expression levels, determined by immunoblotting, in MOLM13 versus midostaurin-resistant MOLM13 cells. ImageJ 32 software was used for densitometry. Briefly, to get band intensities, the area of all the bands was first measured, and then normalized to the control lane, and then normalized to total protein.

We sought to confirm that potentiation of midostaurin by dual FLT3/SYK inhibition and targeted SYK inhibition could be advantageous in terms of overriding some forms of drug resistance. Midostaurin-resistant MOLM13-luc+ cells were previously established via prolonged exposure to midostaurin, and exhibited increased levels of cell surface FLT3 protein and elevated levels of phospho-MAPK, however normal levels of phospho-STAT5 [28]. Midostaurin-resistant MOLM13-luc+ cells were tested with combinations of midostaurin and R406, R788, or PRT062607, and nearly additive effects were observed between midostaurin +/− R406 or R788 against the drug-resistant cells across a range of concentrations (Figure 10A-10B and Table 2). Positive combination effects between midostaurin and PRT062607 against these cells were more striking, with combination indices indicative of synergy (Figure 10C and Table 2). Midostaurin-resistant MOLM13-luc+ cells are less responsive to midostaurin than parental MOLM13-luc+ as evidenced by a rightward shift in the midostaurin dose-response curve (Figure 10D). Drug resistance is likely due to the over-expression of FLT3 protein in the midostaurin-resistant cells, as previously reported and also confirmed in the present study (Figure 10E). SYK protein expression, however, remained the same between the two lines (Figure 10E).

**Figure 11:**
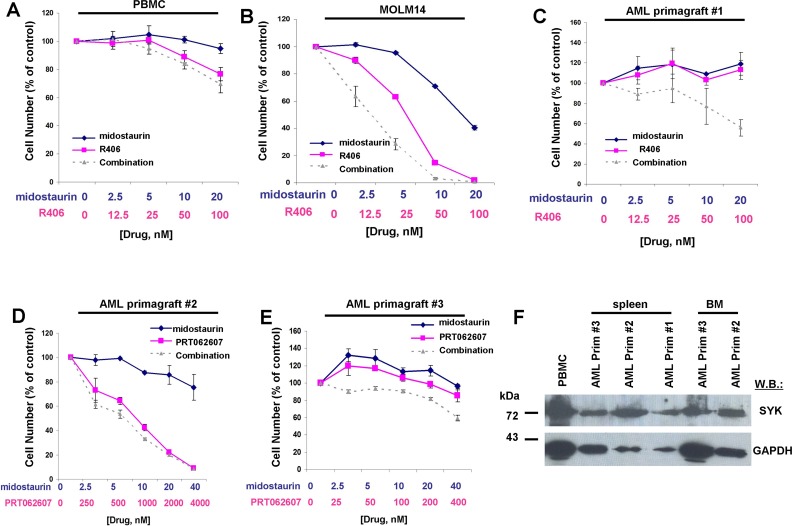
Potentiation of effects of midostaurin against drug-resistant AML primagraft cells by R406 and PRT062607 **A**.-**B**. Three-day treatment of normal PBMCs (A) with midostaurin+/−R406. MOLM14 cells (B) were treated in parallel as a positive control for drug effectiveness. **C**.-**E**. Three-day treatment of mutant FLT3-positive AML primagrafts with midostaurin+/−R406 (C) and midostaurin+/−PRT062607 (D-E). **F**. SYK protein expression in normal PBMC cells and FLT3-ITD-positive AML primagraft cells.

We next evaluated the therapeutic potential of combining midostaurin with dual SYK/FLT3 or SYK inhibitors by testing the resulting growth inhibitory effect on FLT3-ITD-expressing AML primagraft cells from relapsed patients or patients that were refractory to chemotherapy treatment. As normal controls, PBMC cells from a healthy donor were tested with the combination of midostaurin+R406, and little to no inhibitory effects of the agents alone or combined were observed (Figure 11A). MOLM14 cells were tested in parallel with the normal PBMC cells as a positive control for drug activity (Figure 11B). We then tested the ability of R406 to potentiate midostaurin (Figure 11C). Whereas little to no single agent activity was observed for midostaurin at up to 20 nM or R406 at up to 100 nM against AML primagraft sample #1 (disease stage at time of sample acquisition: relapsed post-allogeneic HSCT, Supplementary Table 1), the combination of midostaurin and R406 led to a higher percentage of cell death as compared to either single agent across the full range of concentrations (Figure 11C). A second AML primagraft sample (#2) (disease stage at time of sample acquisition: relapsed after multiple modalities, Supplementary Table 2) was tested with midostaurin+/−PRT062607 at concentrations previously tested against FLT3-ITD-expressing cell lines (Figure 11D). Whereas little to no single agent activity was observed for midostaurin at up to 40 nM, PRT062607 treatment led to around 50% cell death at 1000 nM, and a slight leftward shift in the dose-response curve (indicating a positive drug combination effect) was observed for the drug combination (Figure 11D). Interestingly, treatment of a third AML primagraft sample (#3) (disease stage at time of sample acquisition: primary refractory post-induction, Supplementary Table 3) with midostaurin (up to 40 nM), alone and in combination with PRT062607 across a comparatively conservative concentration range (up to only 400 nM, a 10-fold lower concentration than was used against FLT3-ITD-positive cell lines), resulted in a higher percentage in the combination of agents as compared to either agent alone (Figure 11E). SYK protein expression was detectable in all FLT3-ITD-positive AML primagrafts tested; SYK expression in bone marrow and spleen samples corresponding to the primagrafts is shown alongside normal PBMC cells (Figure 11F).

### Potentiation of the effects of crenolanib by targeted SYK suppression against cells expressing FLT3-ITD and activated SYK

**Figure 12:**
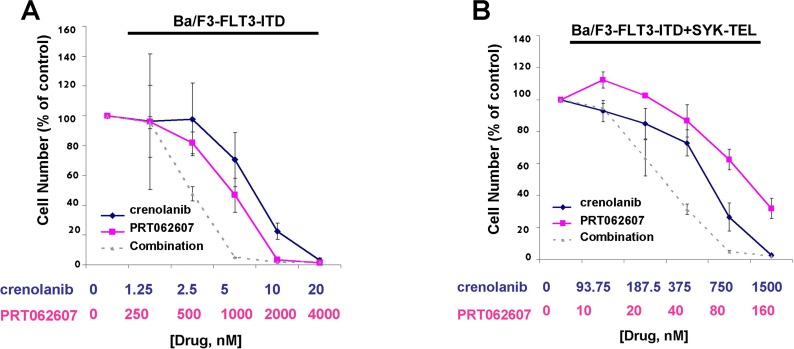
Potentiation of effects of crenolanib by PRT062607 against Ba/F3-FLT3-ITD and Ba/F3-FLT3-ITD+SYK-TEL cells **A**.-**B**. Three-day treatment of Ba/F3-FLT3-ITD (A) or Ba/F3-FLT3-ITD+SYK-TEL (B) cells with crenolanib+/−PRT062607. Error bars represent the mean+/−S.D. for all proliferation studies.

Given that dual SYK/FLT3 inhibition and targeted SYK inhibition potentiated the effects of midostaurin, an established dual inhibitor of SYK and FLT3, against cells driven by FLT3-ITD, activated SYK, or a combination of both, we were interested in investigating the ability of additional SYK inhibition to similarly augment the effectiveness of targeted FLT3 inhibition. The combination of crenolanib with PRT062607 was nearly additive against Ba/F3-FLT3-ITD cells for the concentrations of inhibitors at which 25% and 50% of cell growth inhibition is observed (ED25-ED50) (Figure 12A and Table 2). PRT062607 potentiated the anti-leukemic effects of crenolanib against cells co-expressing FLT3-ITD+SYK-TEL, with nearly additive effects observed for concentrations of inhibitors at which 25%, 50%, 75%, and 90% of cell growth inhibition is observed (ED25-ED90) (Figure 12B and Table 2).

## DISCUSSION

SYK has been shown to be important in FLT3 mutant-positive AML in that it transactivates FLT3-ITD, and through over-expression has been shown to play a role in AML transformation and resistance to FLT3 inhibition [10]. In addition, the combined inhibition of FLT3 by a targeted FLT3 inhibitor (quizartinib) and SYK has been demonstrated to be more effective than FLT3 inhibition alone [10]. Midostaurin and its metabolites, CGP52421 and CGP62221, have been reported as inhibitors of SYK in advanced systemic mastocytosis [25]. However, the activity of midostaurin as an inhibitor of SYK has not yet been explored in FLT3-mutant-positive AML.

Midostaurin, which has recently been FDA approved, was shown in a large trial (RATIFY (CALGB 10603)) in newly diagnosed patients to increase survival when combined with standard chemotherapy [7]. This study supports the notion that inhibition of FLT3 is important in patients with mutations in the FLT3 gene, and also highlights the clinical effectiveness of midostaurin as a therapeutic for AML. As highly activated SYK has been found to occur at a higher frequency in AML patients harboring the *ITD* mutant than patients expressing wt *FLT3* [10], we were interested in exploring the activity of midostaurin against SYK using cell-based models of SYK-driven and SYK- and FLT3-driven leukemia. We found that while midostaurin is an excellent inhibitor of FLT3-ITD-expressing cells, it is a relatively weak inhibitor of activated SYK alone (SYK-TEL or TEL-SYK) and activated SYK (SYK-TEL) co-expressed with FLT3-ITD. Specifically, we found the potency of midostaurin against activated SYK-expressing cells to be 2.5 to 5-fold less than that of the targeted SYK inhibitor, PRT062607. Conversely, the potency of midostaurin against cells expressing FLT3-ITD or FLT3-ITD+TEL-SYK (which are believed to be driven predominantly by FLT3-ITD due to a lack of transactivation of FLT3 by TEL-SYK) was close to 100-fold higher than PRT062607. Thus, the benefit of dual suppression of FLT3 and SYK by midostaurin allows this agent to be more versatile than a targeted SYK inhibitor in that it is effective against cells expressing oncogenic FLT3, activated SYK, or cells expressing both oncoproteins.

Having established the extent by which SYK is inhibited by midostaurin in addition to FLT3 in comparison to the dual SYK/FLT3 inhibitors, R406 and R788, we were then interested in exploring whether or not additional SYK inhibition is able to further improve the efficacy of midostaurin. Potentiation of the effects of midostaurin by R406 or PRT062607, as measured in cell growth assays and evidenced by increased killing of constitutively activated SYK and/or mutant FLT3-expressing cells, correlated with increased suppression of signaling molecules, including those involved in the phosphatidylinositol 3-kinase pathway and mitogen-activated protein kinase signaling, characterized as phosphorylated substrates and exerting effects downstream of either oncogenic FLT3 or activated SYK [29, 30, 5, 24]. Our results are consistent with earlier findings showing that, in a SYK-TEL cooperative model with FLT3-ITD, the combination of PRT062607 and quizartinib increased survival of mice, which correlated with inhibition of FLT3-ITD and SYK activation [10]. Importantly, our results also suggest that the effects of midostaurin can be potentiated by dual FLT3/SYK suppression or more targeted SYK suppression in the context of midostaurin-resistant leukemia and in FLT3-ITD-expressing primary cells.

Drug combination effects were observed against phosphorylated STAT5, S6, MAPK, and AKT by midostaurin+R406 or midostaurin+PRT062607 in cells expressing constitutively activated SYK. Significantly, STAT5 has been identified as a major downstream signaling mediator of constitutively activated SYK, both *in vitro* and *in vivo*, and targeted deletion of Stat5 *in vivo* was shown to fully abrogate AML caused by TEL-SYK [24]. These results support the notion of STAT5 as a key driver of SYK-induced cellular transformation, and shed light on the potential clinical importance of our observation of enhanced suppression of activated STAT5 by midostaurin combined with inhibitors of FLT3 and SYK or SYK alone.

Importantly, the efficacy of midostaurin, R406 and R788 toward cells expressing FLT3-ITD alone or FLT3-ITD with activated SYK was compared with the efficacy of targeted FLT3 inhibitors quizartinib and crenolanib, neither of which targets SYK. We observed that all of the inhibitors were highly potent toward FLT3-ITD-driven cells in the absence of activated SYK, however midostaurin, R406 and R788 showed relatively higher potency toward cells expressing activated SYK. Whereas inhibition of SYK alone does not substitute for FLT3 inhibition and is incapable of inducing death of cells predominantly driven by FLT3-ITD, additional SYK inhibition was observed to potentiate the anti-proliferative activity of both multi-targeted and selective FLT3 inhibitors against cells driven by activated SYK. Taken together, the ability of SYK and dual FLT3/SYK suppression to enhance the growth inhibitory effects of midostaurin and other FLT3 inhibitors in the context of both kinase inhibitor-sensitive and kinase inhibitor-resistant disease warrants further investigation for the continued development and optimization of midostaurin as a treatment strategy for AML.

## MATERIALS AND METHODS

### Chemical compounds and biologic reagents

Midostaurin was synthesized by Novartis Pharma AG, Basel, Switzerland. R406, R788, PRT062607, crenolanib and quizartinib were purchased from Selleckchem (Houston, TX) and dissolved in DMSO to obtain a 10 mM stock solution. Serial dilutions were then made, to obtain final dilutions for cellular assays with a final concentration of DMSO not exceeding 0.1%.

### Cell lines and cell culture

Ba/F3 (interleukin [IL]-3-dependent murine pro-B) cells engineered to express FLT3-ITD, SYK-TEL, TEL-SYK, FLT3-ITD+SYK-TEL, and FLT3-ITD+TEL-SYK [10] were provided by Dr. Kimberly Stegmaier. Ba/F3 cells were engineered to over-express wt FLT3 as previously described [6].

Human AML-derived, FLT3-ITD-expressing MV4,11 cells were obtained from Dr. Anthony Letai. The human AML-derived, FLT3-ITD-expressing line, MOLM14 [31], was provided to us by Dr. Scott Armstrong, Dana-Farber Cancer Institute (DFCI), Boston, MA.

The human AML-derived, FLT3-ITD-expressing cell line, MOLM-13 (DSMZ (German Resource Centre for Biological Material), was engineered to express luciferase fused to neomycin phosphotransferase (pMMP-LucNeo) by transduction with a VSVG-pseudotyped retrovirus as previously described [32]. Development of PKC412-resistant cells derived from long-term culture or drug-resistant colonies (MOLM13-R-PKC412 (CFU)) was described previously [28].

All cell lines used in this study were cultured with 5% CO_2_ at 37°C, at a concentration of 2×10^5^ to 5×10^5^ in RPMI (Mediatech, Inc., Herndon, VA) with 10% fetal bovine serum (FBS) and supplemented with 2% L-glutamine and 1% penicillin/streptomycin. Parental Ba/F3 cells and Ba/F3-wt FLT3 cells were cultured in RPMI with 10% FBS and supplemented with 2% L-glutamine and 1% penicillin/streptomcyin, as well as 20% WEHI (as a source of IL-3).

Human cell lines were submitted for cell line authentication and were authenticated within 6 months of manuscript preparation through cell line short tandem repeat (STR) profiling (DDC Medical, Fairfield, OH and Molecular Diagnostics Laboratory, Dana-Farber Cancer Institute). All cell lines tested matched >80% with lines listed in the ATCC or DSMZ Cell Line Bank STR. All cell lines were confirmed to be virus- and *Mycoplasma*-free.

### Immunoblotting and immunoprecipitation

Protein lysate preparation, immunoblotting, and immunoprecipitation were carried out as previously described [6]. Briefly, to obtain protein lysates for immunoprecipitation and immunoblotting, cells were lysed in lysis buffer (0.02 M Tris [pH 8.0], 0.15 M NaCl, 10% glycerol, 1% NP-40 (wt/vol), 0.1 M NaF, 1 mM phenylmethylsulfonyl fluoride (PhCH2SO2), 1 mM sodium orthovanadate (Na3VO4), and HALT^TM^ protease inhibitor cocktail, EDTA-free (100X) (ThermoFisher Scientific, Waltham, MA). Protein lysates were incubated on ice for 25 min, vortexed at 5 min intervals, and then centrifuged for 15 min at 12,000 X g. Supernatants were saved, and the Bio-Rad Protein Assay was used to determine protein yields (Bio-Rad Laboratories, Hercules, CA). Equivalent amounts of protein were then loaded onto a gel for immunoblotting. For immunoprecipitation, cell lysates were incubated with antibody and protein A/G Sepharose overnight with rocking at 4°C. After the incubation, immune complexes were washed twice with lysis buffer, twice with 1X PBS, and were then resuspended in Laemmeli's sample buffer and boiled for 5 min. For immunoblotting and immunoprecipitation, samples were resolved on a sodium dodecyl sulfate (SDS)-10% polyacrylamide gel. Then proteins were electrophoretically transferred to a Protran nitrocellulose transfer and immobilization membrane (Schleicher and Schuell, Dassel, Germany). The membrane was then blocked overnight at 4°C with BSA or milk in 1X TBS (10mM Tris-HCl [pH 8.0], 150 mM NaCl) and then probed with antibody overnight at 4°C in 1X TBST buffer (10 mM Tris-HCl [pH 8.0], 150 mM NaCl, 0.05% Tween20). After three washes with 1X TBST, membranes were incubated for 1 hr at 25°C with anti-mouse immunoglobulin (horseradish peroxidase-linked whole antibody from sheep) (Amersham Life Science, Inc., Arlington Heights, IL). The membrane was washed 5X in 1X TBST buffer, with 5 min intervals between buffer changes, and bound antibody was detected with enhanced luminol and oxidizing reagent as specified by the manufacturer (NEN Life Science Products, Boston, MA).

### Antibodies

The following antibodies were purchased from Cell Signaling Technology (Danvers, MA): total AKT (rabbit, #9272) and total p44/42 MAPK (Erk1/2) (3A7) (mouse, #9107) were used at 1:1000. Anti-GAPDH (D16H-11) XP (R) (rabbit mAb, #5174) was used at 1:1000. Phospho-AKT (Ser 473) (D9E) XP(R) (rabbit mAb, #4060) was used at 1:1000. Phospho-p44/42 MAPK (T202/Y204) (rabbit, #9101) was used at 1:1000. Phospho-S6 ribosomal protein (S235/236) (D57.2.2E) XP (R) (rabbit mAb, #4858) was used at 1:1000. Total S6 ribosomal protein (SG10) (rabbit mAb, #2217) was used at 1:1000. Phospho-STAT5 Tyr694 (rabbit, #9351S) and total STAT5 (3H7) (rabbit, #9358 mAb) were used at 1:1000. The Syk (D3Z1E) XP (rabbit mAb, #13198) was used at 1:1000.

FLT3/Flk-2 (C-20) (sc-479) was purchased from Santa Cruz Biotechnology, Inc., (Dallas, TX) and used at 1:1000 for immunoblotting. Anti-pTyr (mouse, clone 4G10) was purchased from Upstate Biotechnology (Lake Placid, NY) and was used at 1:1000 in the presence of 4% BSA.

### Cell growth studies

The trypan blue exclusion assay has been previously described [6] and was used for quantification of cells prior to seeding for Cell Titer Glo assays. The CellTiter Glo assay (Promega, Madison, WI) was used for proliferation studies and carried out according to manufacturer instructions. Cell viability is reported as percentage of control (untreated) cells, and error bars represent the standard deviation for each data point.

### Apoptosis assays and cell cycle analysis

Programmed cell death of inhibitor-treated cells was determined using the Annexin-V-Fluos Staining Kit (Boehringer Mannheim, Indianapolis, IN), as previously described [6]. Briefly, cells were washed once with 1X PBS and pelleted by centrifugation for 5 minutes at 1500 rpm. Cells were resuspended in 100μl of 20% propidium iodide (PI) and 20% Annexin-V-fluorescein labeling reagent, either agent alone (as controls), or were left unstained by diluting only in 1X binding buffer (as a control). All samples were incubated for 10-15 minutes at room temperature, and then stained cells were diluted in 0.8 mL of 1X binding buffer. Cells were then analyzed by flow cytometry.

Cell cycle analysis was performed as previously described [6]. Briefly, around 500,000 cells were centrifuged at 1500 rpm for 5 min and washed in 1X PBS, and the pellet was resuspended in 500 μl of propidium iodide solution (50 μg/ml propidium iodide, 0.1% NP-40, 0.1% sodium citrate). The mixture was stored in the dark at 4°C for a minimum of 15 min, and then analyzed by flow cytometry.

### Caspase 3 activation assay

For measurement of caspase-3 and -7 activity, the Apo-ONE Homogenous Caspase-3/7 Assay kit was used (Promega, Madison, WI). The assay was carried out according to manufacturer's instructions.

### Drug combination studies

For drug combination studies, cell viability was first determined using the Trypan Blue exclusion assay to quantify cells for cell seeding, and Cell Titer Glo was then implemented for proliferation studies. Single agents were added simultaneously at fixed ratios to cells. Cell viability was expressed as the function of growth affected (FA) drug-treated versus control cells; data were analyzed by Calcusyn software (Biosoft, Ferguson, MO and Cambridge, UK), which was utilized for synergy measurement and based on isobologram generation and the method of Chou-Talalay (1984) [33]. This method utilizes the median effect principle to quantify the effects of drug combinations to determine whether they give greater effects together than expected from a simple summation of their individual effects. After determining the ED_50_ or IC_50_ of each drug, combinations are studied where the concentrations are multiples, or fractions, of the ED/IC_50_. Statistical analysis is automatically part of the computations. Combination indices, values generated by the Calcusyn software, which are less than one indicate synergy, whereas values greater than one indicate antagonism. Calcusyn combination indices can be interpreted as follows: CI <0.1 indicate very strong synergism; values 0.1-0.3 indicate strong synergism; values 0.3-0.7 indicate synergism; values 0.7-0.85 indicate moderate synergism; values 0.85-0.90 indicate slight synergism; values 0.9-1.1 indicate nearly additive effects; values 1.10-1.20 indicate slight antagonism; values 1.20-1.45 indicate moderate antagonism; values 1.45-3.3 indicate antagonism; values 3.3-10 indicate strong antagonism; values >10 indicate very strong antagonism.

## SUPPLEMENTARY MATERIALS FIGURES AND TABLES








