# Supplementary material for: Characterization of midostaurin as a dual inhibitor of FLT3 and SYK and potentiation of FLT3 inhibition against FLT3-ITD-driven leukemia harboring activated SYK kinase
Source: Oncotarget. 2017 Jul 6;8(32):52026–44. doi: 10.18632/oncotarget.19036 (PMC5581010; doi:10.18632/oncotarget.19036)
Supplement: Supplementary file 1 [file oncotarget-08-52026-s001.pdf]

Characterization of midostaurin as a dual inhibitor of FLT3 and SYK and potentiation of FLT3 inhibition against FLT3-ITD-driven leukemia harboring activated SYK kinase

SUPPLEMENTARY MATERIALS

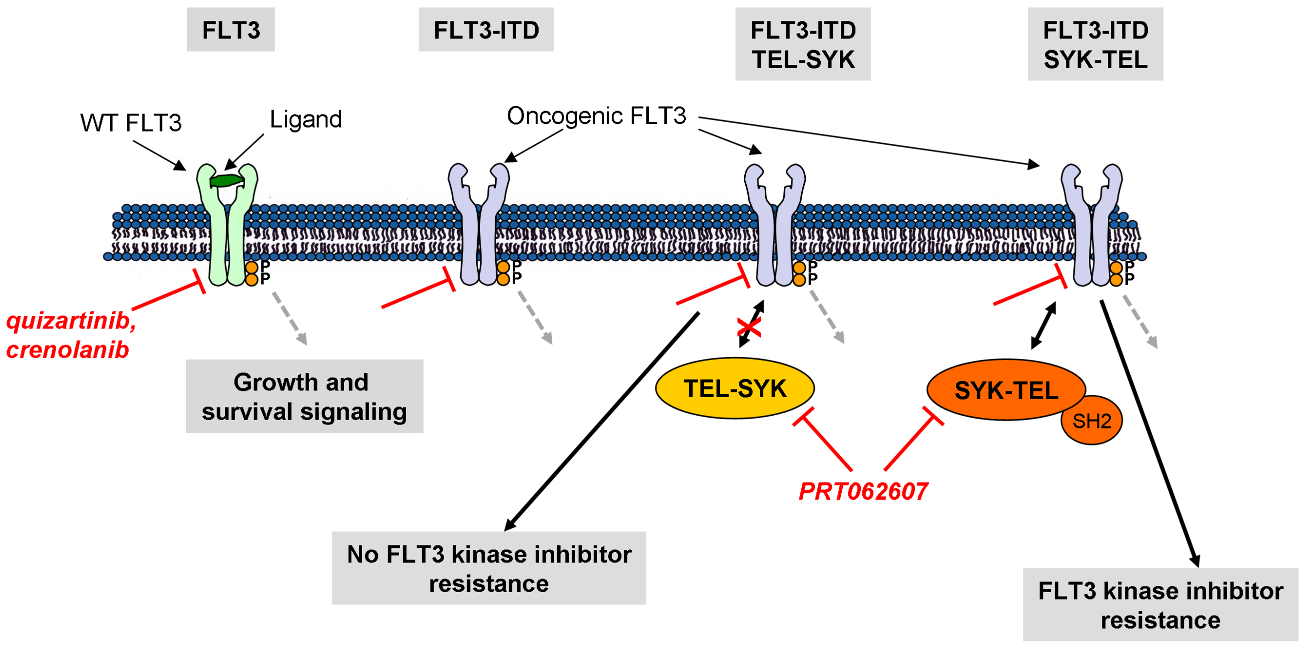

Supplementary Figure 1: Cartoon representation of TEL-SYK and SYK-TEL signaling.

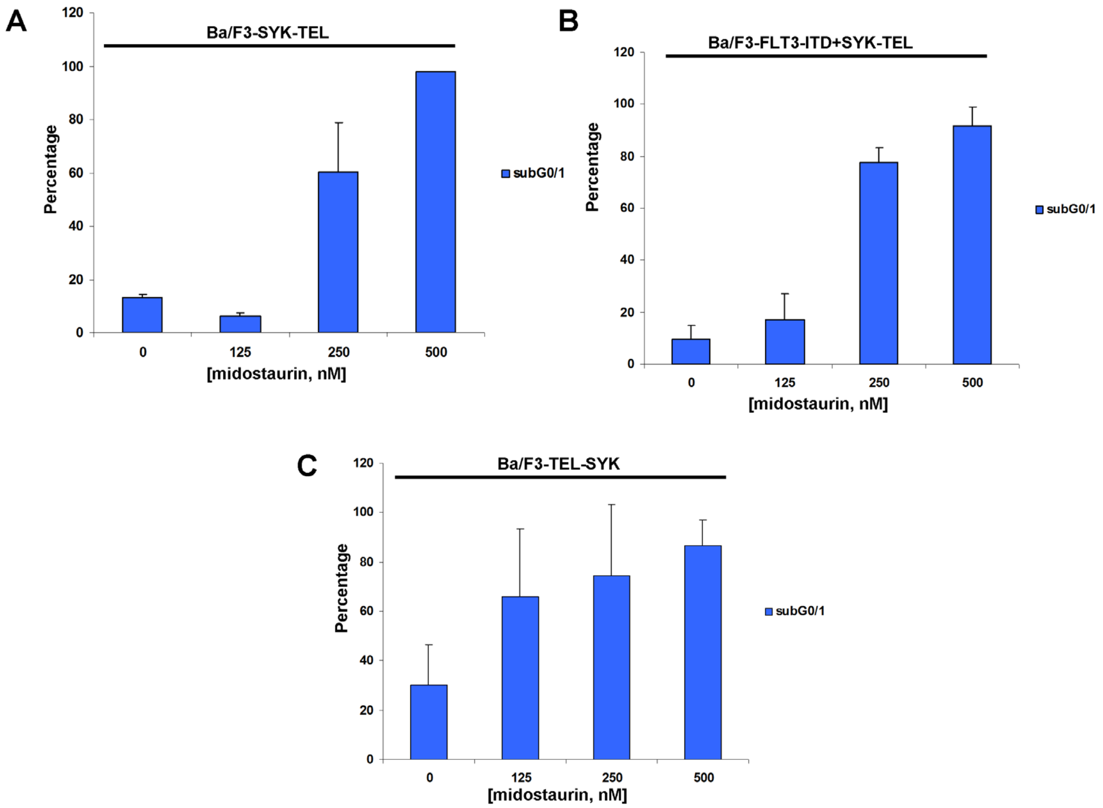

Supplementary Figure 2: (A-C) Midostaurin treatment of active SYK-expressing cells leads to an increased percentage of cells in subG0/G1, as determined by PI staining for cell cycle analysis; this correlates with an increase in apoptotic cells with midostaurin treatment as determined by annexin/pi staining.

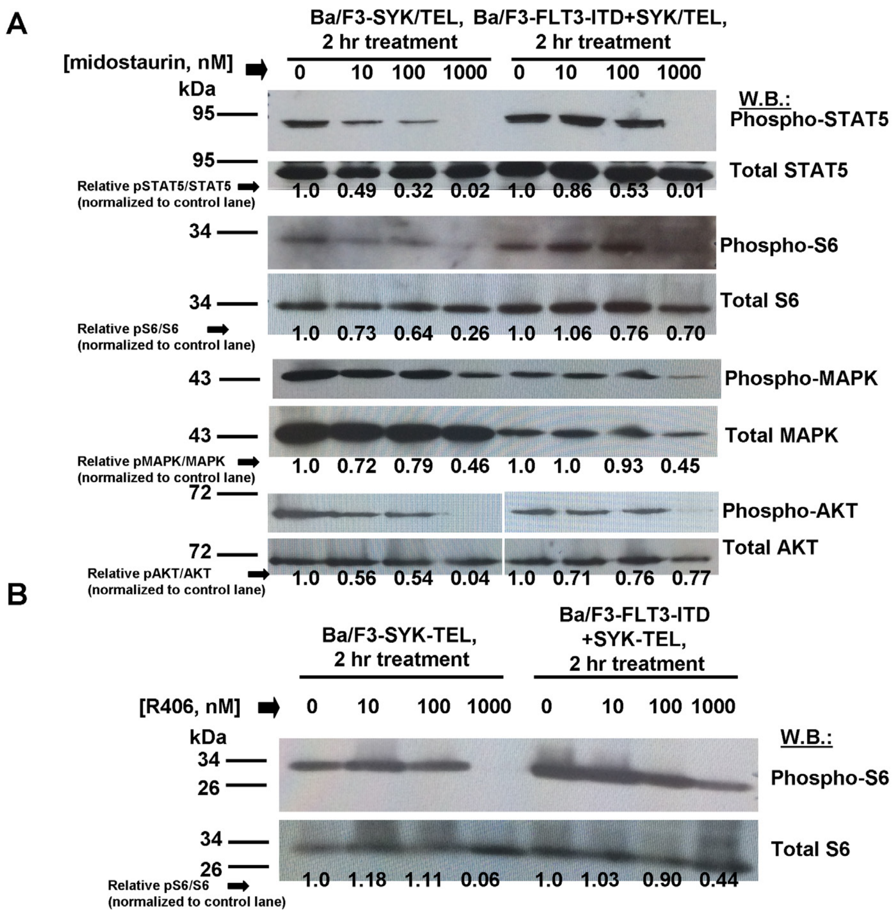

Supplementary Figure 3: Effects of midostaurin (A) and R406 (B) on downstream effectors of SYK-TEL and FLT3-ITD+SYK-TEL. ImageJ 32 software was used for densitometry. Briefly, to get band intensities, the area of all the bands was first measured, and then normalized to the control lane, and then normalized to total protein.

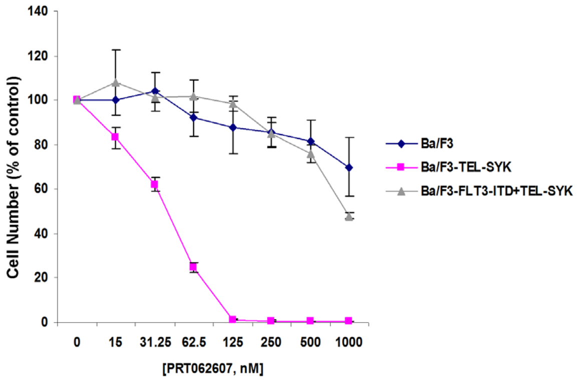

Supplementary Figure 4: Effects of PRT062607 on inhibition of proliferation of parental Ba/F3, Ba/F3-TEL-SYK and Ba/F3-FLT3-ITD+TEL-SYK cells. Approximately 3-day treatment of Ba/F3 cells (in the presence of 20% WEHI, used as a source of IL-3), Ba/F3-TEL-SYK cells (-WEHI), and Ba/F3-FLT3-ITD+TEL-SYK cells (-WEHI) with PRT062607.

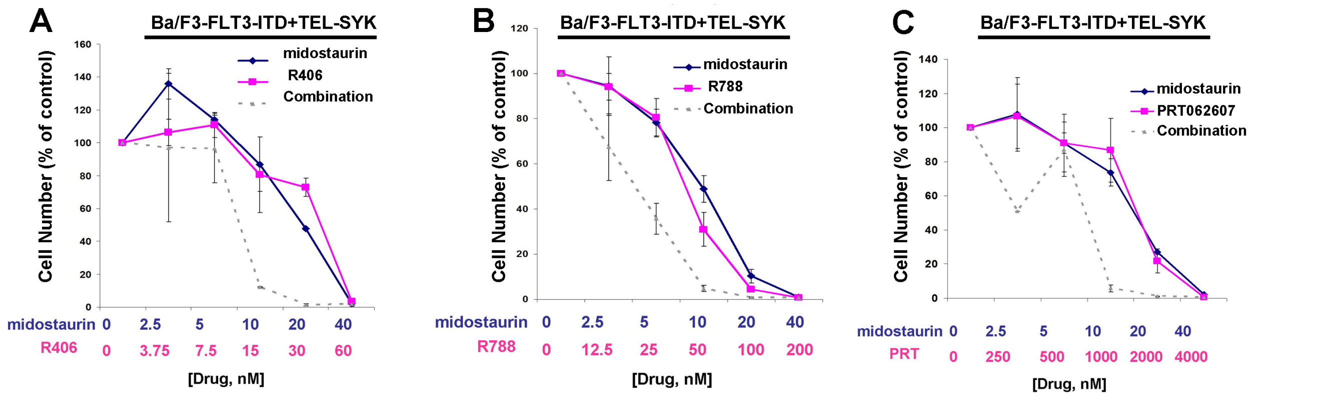

Supplementary Figure 5: Potentiation of effects of midostaurin against Ba/F3-FLT3-ITD+TEL-SYK cells by R406, R788, and PRT062607. (A-C) Approximately 3-day treatments of Ba/F3-FLT3-ITD+TEL-SYK cells with midostaurin+/-R406, midostaurin+/-R788, or midostaurin+/-PRT062607.

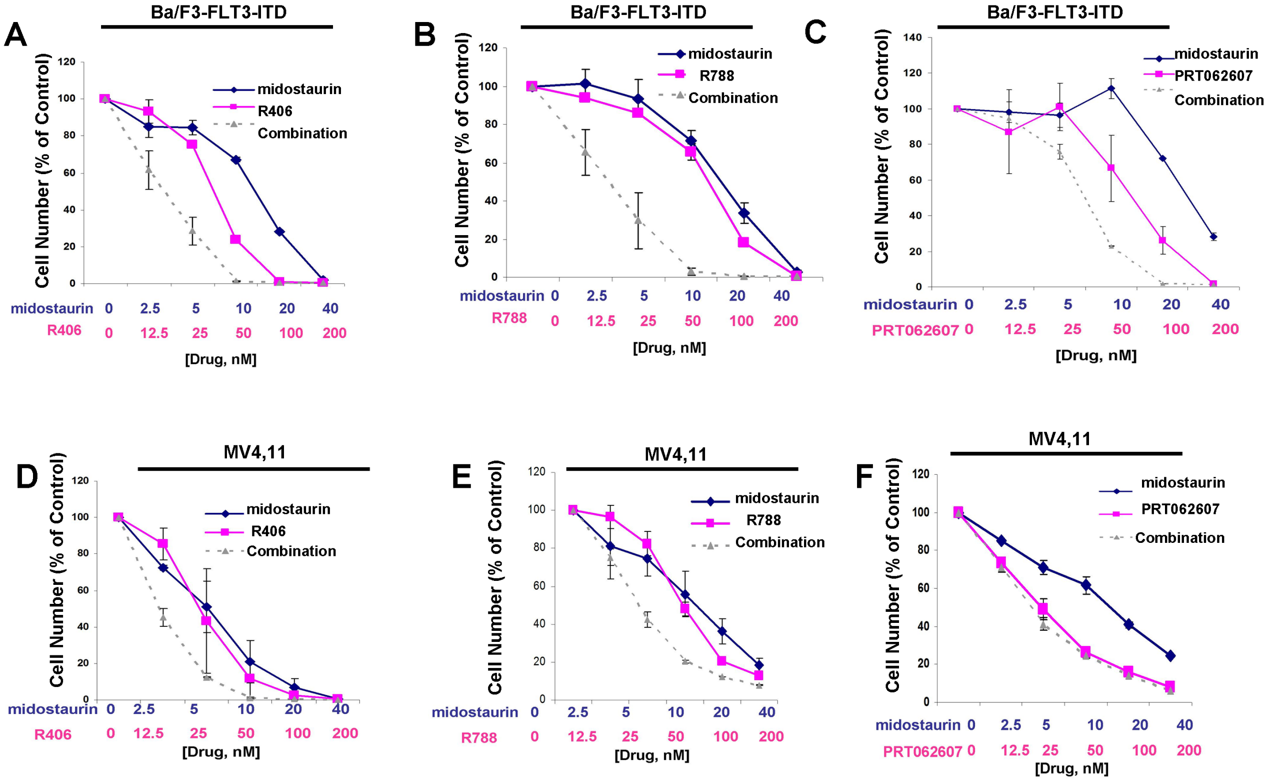

Supplementary Figure 6: Potentiation of midostaurin by R406, R788 and PRT062607 against FLT3-ITD-driven cell lines. (A-C) Approximately 3-day treatments of Ba/F3-FLT3-ITD cells with midostaurin+/-R406, midostaurin+/-R788, or midostaurin+/-PRT062607. (D-F) Approximately 3-day treatments of MV4,11 cells with midostaurin+/-R406, midostaurin+/-R788, or midostaurin+/-PRT062607.

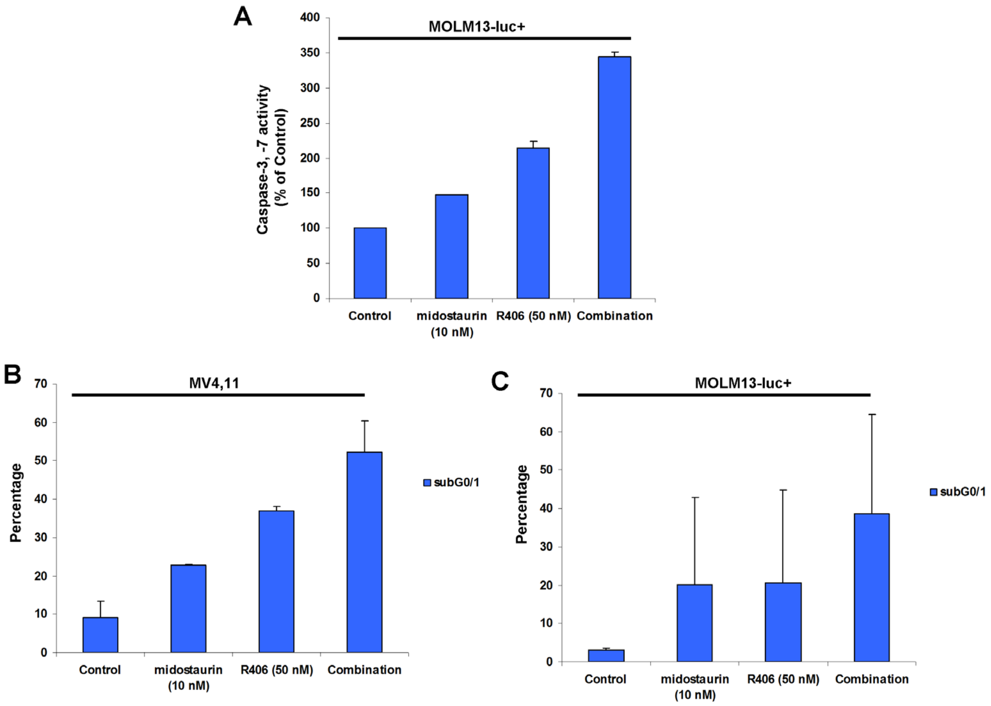

Supplementary Figure 7: Combination treatment with midostaurin and dual SYK/FLT3 kinase inhibitors leads to increased apoptosis as compared to single agent treatments. (A) Caspase-3 and -7 activity in MOLM13-luc+ cells treated with midostaurin (10 nM), R406 (50 nM), or a combination. (B-C) Percentage of subG0 cells as determined by PI staining for cell cycle analysis in MV4,11 cells (B) and MOLM13-luc+ cells (C) treated with midostaurin+/- R406 and R788, respectively.

For Supplementary Tables see in Supplementary Files
