# Supplementary material for: Characterization of midostaurin as a dual inhibitor of FLT3 and SYK and potentiation of FLT3 inhibition against FLT3-ITD-driven leukemia harboring activated SYK kinase
Source: Oncotarget. 2017 Jul 6;8(32):52026–44. doi: 10.18632/oncotarget.19036 (PMC5581010; doi:10.18632/oncotarget.19036)
Supplement: Supplementary file 2 [file oncotarget-08-52026-s002.doc]

| **Supplementary Table 1: Patient information for FLT3-ITD-positive AML primagraft #1** |
| --- |
| **Pathologic diagnosis:** AML M4/M5  **WHO classification:** AML with recurrent gene mutations |
| **Disease stage at time of sample acquisition:** Relapsed post-allogeneic HSCT |
| **Age, gender:** 61, male |
| **Percent tissue involvement:** 71 |
| **Notable clinical features:** Prior prostate cancer s/p prostatectomy/EBRT 26 months prior as well as papillary thyroid cancer for which he was being treated with RAI at the time of the AML diagnosis; of note the relapse AML now expresses CD19 which is atypical. |
| **Patient clinical details:** Relapsed following 6 +3, consolidation HiDAC, allogeneic HSCT in CR1. |
| **Source tumor karyotype:** 47,X,-Y,del(6)(q15q21),+8,+14[15]/47,idem,t(1;9)(q23;q34)[4]//46,XX[1]  **Source karyotype simplified:** Trisomies 8 and 14, deletion 6q, and loss of Y chromosome; 3 metaphases also contained t(1;9). |
| **FISH positive:** nuc ish(DXZ1x1)[161/200]//(DXZ1x2)[39/200] |
| **Immunophenotype positive:** CD45(dim), HLA-DR, CD13, CD33, CD117 (subset), CD15 (subset), CD19 (aberrant) and CD7 (aberrant dim) |
| **Immunophenotype negative:** CD34, CD10, CD20, CD79a, cytoplasmic CD22, and other monocytic, B and T lymphoid markers |
| **Presenting WBC:** 260000 |
| **Molecular alterations (FLT3):** FLT3-ITD  c.2503C>A p.D835Y - in 43.0% of 1767 reads |
| **Molecular alterations (NMP1):** c.859_860insTCTG p.W288fs*>9 - in 38.1% of 412 reads |
