# Supplementary material for: Characterization of midostaurin as a dual inhibitor of FLT3 and SYK and potentiation of FLT3 inhibition against FLT3-ITD-driven leukemia harboring activated SYK kinase
Source: Oncotarget. 2017 Jul 6;8(32):52026–44. doi: 10.18632/oncotarget.19036 (PMC5581010; doi:10.18632/oncotarget.19036)
Supplement: Supplementary file 3 [file oncotarget-08-52026-s003.doc]

| **Supplementary Table 2: Patient information for FLT3-ITD-positive AML primagraft #2** |
| --- |
| **Pathologic diagnosis:** AML arose from RAEB2 MDS |
| **Disease stage at time of sample acquisition:** Relapse after multiple modalities |
| **Age, gender, race:** 59, female, caucasion |
| **Percent tissue involvement:** Not reported |
| **Notable clinical features:** None listed |
| **Patient clinical details:** Achieved remission s/p induction 7+3 and underwent transplant in CR1 then relapsed |
| **Source tumor karyotype:** None reported  **Source karyotype simplified:** None reported |
| **FISH negative:** None reported |
| **Immunophenotype positive:** No report |
| **Immunophenotype negative:** No report |
| **Presenting WBC:** No report |
| **Molecular alterations (FLT3):** FLT3-ITD |
| **Molecular alterations (NMP1):** None reported |
