# Supplementary material for: Characterization of midostaurin as a dual inhibitor of FLT3 and SYK and potentiation of FLT3 inhibition against FLT3-ITD-driven leukemia harboring activated SYK kinase
Source: Oncotarget. 2017 Jul 6;8(32):52026–44. doi: 10.18632/oncotarget.19036 (PMC5581010; doi:10.18632/oncotarget.19036)
Supplement: Supplementary file 4 [file oncotarget-08-52026-s004.doc]

| **Supplementary Table 3: Patient information for FLT3-ITD-positive AML primagraft #3** |
| --- |
| **Pathologic diagnosis:** AML M5a  **WHO classification:** AML with recurrent gene mutations |
| **Disease stage at time of sample acquisition:** Primary refractory post-induction |
| **Age, gender:** 9.1, pediatric, male |
| **Percent tissue involvement:** Not reported |
| **Notable clinical features:** None listed |
| **Patient clinical details:** M5 monoblastic AML w/ FLT3-ITD; day 22 of induction I (refractory); WBC on presentation 0.72 (ANC: 0.05, Platelets 38); treated as per DFCI 04-172 w/ daunorubicin, etoposide, and low dose Ara-C; bone marrow on 3/12/2014 with 13% bone marrow involvement by flow |
| **Source tumor karyotype:** 46,XY,add(6)(q21),add(9)(p24)[10]/46,XY[10]  **Source karyotype simplified:** 9p and 6q additional material |
| **FISH negative:** Rearrangement or loss/gain of MLL, CBFB rearrangement, RUNX1T1/RUN1 (ETO/AML1) rearrangement, PML/RARA translocation. nuc ish(RUNX1T1,MLL,PML,CBFB,RARA,RUNX1)x2[500] |
| **Immunophenotype positive:** CD45 (intermediate), CD117, CD34 , CD13, and CD33 |
| **Immunophenotype negative:** No report |
| **Presenting WBC:** 720 |
| **Molecular alterations (FLT3):** FLT3-ITD |
| **Molecular alterations (NMP1):** None reported |
